# Looking Forward

**Published:** 1890-02

**Authors:** Rodrigues Ottolengui

**Affiliations:** 115 Madison Avenue, N. Y.


THE
AMERICAN JOURNAL
—OF—
DENTAL SCIENCE.

VOL. XXIII. BALTIMORE, FEBRUARY, 1890. NO. 10.

ARTICLE I.

### LOOKING FORWARD.

DENTISTRY IN THE YEAR 2000—THE COLLEGE SYSTEM—THE EVOLUTION OF THE DENTAL PROTECTIVE ASSOCIATION, AND THE STATUS OF THE AMERICAN DENTAL TRADE ASSOCIATION—THE REPRESENTATIVE DENTAL JOURNAL—PROGRESS IN METHODS.

Dreamers of all ages have been ridiculed, and yet it is to these men of imagination that much of the progress of the world is due. The prophecy that a thing will be, not infrequently is the initial impetus toward accomplishment, and the prophet is credited with prevision, whereas his dream would not have been realized if he had not related it.

For this reason I shall give you in detail a remarkable vision of the future of our profession, which has been my dream.

The old year was dying; a new one was approaching. I was lying down, meaning to rest rather than to sleep.

Thought, which bridges time, carried me hurriedly over the past of my professional career. Brief thought it was, I could mark many distinct landmarks of progress. With the dawning of the new year I not unnaturally speculated as to the future. I "looked forward."

I have no recollection of having fallen asleep, but suddenly it seemed to me that I was on a train of cars. Whither bound I knew not, nor did I seem to care. I became aware of the fact that I was among strangers. This I did not note at first, because one does not expect to meet acquaintances on a train; it was made noticeable when I observed that the other men were chatting together as though well acquainted with each other. I listened to the conversation of those nearest to me, and was surprised to find "Dentistry" the topic. One man was saying:

"Yes, I think I shall surprise the convention to-morrow. I shall show what I believe to be a veritable case of Pyorrhœa alveolaris, as the disease was known and described in the journals of a hundred years ago. There has not been a genuine case reported in thirty years, you know."

Could I be hearing aright? Pyorrhœa not known for thirty years? Then surely—— but the next words which reached me fairly startled me:

"Yes, Doctor; undoubtedly your case will prove most interesting. The last case that I know of was that shown by Dr. Richards, in 1962, nearly forty years ago."

I could withhold speech no longer. Turning, I addressed the men behind me:

"Pardon me, gentlemen; but will you give me the date?"

"May first."

"And the year?"

"2,000."

"2,000! You are quite sure?"

"Why, of course. What is the matter with you, Raymond? Are you out of your reckoning?"

"Raymond! Is that your name?"

"Why, Doctor, do you not know yourself? Is anything wrong?"

"Let me think a moment," said I. I was confused. I thought it the year 1890, and these gentlemen declare it to be 2000. I thought my name Ottolengui, and heard myself called Raymond. I pondered deeply, and then spoke:

"Gentlemen, there is a mystery which I can solve but in one way. The Theosophists claim that man lives many lives on this earth in succession, but is ever oblivious of the past. It seems otherwise with me. You know me, and yet I know you not. You tell me it is the year 2000; to me it seems to be the year 1890. If you are correct, then by some mental trick my present has become obscured, and I am conscious only of an existence long since past. Singularly enough, I seem to have been a dentist then as now. I could enlighten you of what to you must seem to be the past, but which to me is as the present."

The two men looked at each other amazed, and then spoke in whispers. I caught the words:

"It will be best to humor him."

"Will you tell me your names?" said I.

"Mine is Harriman, and this is Dr. Ashland, the President of the University."

"The University!" said I. "What University?"

"The National Dental University."

"There was none in my time. We were satisfied with simple colleges."

"You are wrong, Doctor, in your time, since you insist that you do not recognize the present; the profession was not satisfied with the colleges. The University system has grown naturally out of that feeling of dissatisfaction. But Dr. Ashland can tell you about it in detail."

"The facts are thus, began Dr. Ashland: "At first, when dentistry was not recognized as a science, and the practitioner was an artisan with secret methods, the student was practically an apprentice, and learned of a master. Slowly it dawned upon the people that in the New World, America, a *new* and distinctive profession was to be born and thrive. The sphere of the dentist widened, and he took a course of lectures in some medical school. Next the purely dental school was inaugurated, and, in a brief period of years could be found at least one such institution in each large State."

"Yes," said I, "I can remember that there were a large number of colleges, and considerable competition between them. The number of graduates was enormous."

"Exactly. There was great competition. There were too many schools, and the result was that men were graduated who were not sufficiently qualified. The colleges were forced to do this that they might attract students. Finally, the several States began to protect themselves with prohibitory laws. State Boards of Examiners were created with power to confer degrees and to control the practice of dentistry in their localities. Massachusetts, even, made it cumpulsory for a man to obtain her special decree. It was obnoxious for a man graduated by a college to be compelled to submit to a second examination before he was allowed to practice. Yet the law was meant to force a higher standard on the colleges. Thus degrees were multiplied and each State had a separate dental law. In the United State Congress, which met in 1924, a new era dawned. At last it was seen that the sovereignty of the States on certain questions, was a menace to the integrity of the Union as a nation. Amendments to the constitution were recommended, and finally, adopted which enacted national laws on many questions, and repealing the existing State laws. The most important of these laws were in relation to Marriage and Divorce. There was a number of others, and among these were laws regulating the practice of Medicine and Dentistry."

"You mean that there is now a National law regulating the practice of Dentistry?"

"Exactly. It is brief and effective. No man may practice in the United States unless he holds a degree conferred by the National University. That is D. O. S.—Doctor of Oral Surgery. It was deemed advisable to make the degree a new one, that the law could better be enforced."

"But," said I, "Since there can be but one University, it seems unjust to compel attendance from distant localities."

"You do not grasp the idea. The colleges continue to exist as heretofore. They are the schools. A student attends a course covering three full years. If he passes the examination at his College, a certificate is accorded to him which entitles him to attend the University examination."

"He is not admitted at once, then?"

"Of course not. In that case the standard of excellence in the college could not be controlled. No, he must be examined before entering the University. If he passes, he attends a course of lectures covering six weeks, and is then examined for his degree."

"There is still the objection which I raised. The student from a distance is not on an equality with those living near by."

"The certificate granted by the college entitles the student to free passage by rail and return, to and from, the University town. Of course this is simple since the Government bought out and controls all railroads."

"Suppose that a man does not pass either of these examinations?"

"In either case he is returned to the college. And as the college was evidently at fault in granting its certificate to an incompetent man, it is compulsory on them to receive him for a fourth term without fee. This is a further incentive to make the colleges strict."

"The system seems an admirable one. It is odd that it did not occur to those of my time."

"Evolution, my dear doctor, evolution. That is the grand law of all progress. Perfection is aimed at throughout all stages in the ascent. It is not correct to say, 'This was not thought of,' 'That was not conceived.' The highest attainment can be traced back to the smallest beginnings. Each detail was a necessary step. 'Look for instance at the Dental Protective Association.' Possibly its founders would have been horrified if told that their association would ever become a 'patent agency.' Yet so it is."

"The Dental Protective Association a patent agency? Impossible!"

"There again is evolution. It appeared that the association was formed to fight patents, but truly the object was to protect the dentist in his interest in patentable inventions relating to his profession. At first the managers of the enterprise expended their time, money and energy, in the battle against the company which controlled a number of patents. But little was effected. Litigation is tedious. The enactments of one court were overturned in another. They won a suit only to find it again on their hands. The end, however, was inevitable. Time would after all defeat the patent holders. And time was all that did crush them. When this point was reached the association awakened to the fact that if the fighting of patents was its object it would no longer live, since there was no more patents to wage war against."

"What did they do then?"

"What they should have done from the beginning. They announced that they would become a market for dental patents. They would buy all which the Trade Association would recommend."

"Then they had some connection with that body?"

"I will explain that in a moment. In your day the great bug-a-boo was the ethical point that a professional man could not hold a patent. This narrow-minded view more than anything else made it possible for companies to be formed which would buy patents and levy a tax on the profession at large.

When the Protective Association announced that they would buy patents and hold them for the general good of the profession, the question was solved. Men were stimulated toward invention because of the certainty of reaping some pecuniary reward in proportion to their works."

"But how could the society buy patents? What would they do with them?"

"Before I can reply to that question, I must explain the position now held by the Dental Trade Association. About the year 1890 that association attracted considerable attention. The dentists in many sections, claimed that it was a gigantic trust, organized so that the prices of goods might be kept to the highest point. On the other hand, the trade people claimed that they simply co-operated for mutual advantages, and that the dentist gained because the standard of excellence in manufacture was thus kept at a high notch. These two claims were continually set forth by each side, and a long wrangle was kept up, until the Protective Association announced its new departure, after having received the concurrence of all concerned. Once, annually, there is a convention, attended by the Protective Association and the Trade Association. The delegates to this convention comprise one dental delegate to every one hundred dentists who are members of societies, and one trade delegate for every one thousand dollars invested capital."

"What is the object of this convention? And why is it that the dentists who are not in societies have no voice?"

"As to the latter question the reply is manifest; the object is to force the dentists into the societies. This is only just. The dental delegates alluded to comprise the Dental Protective Association which is thus thoroughly representative. Capital is necessary to conduct the business of the association; therefore each delegate is obliged to pay $100 into the treasury of the association, which, you observe, is a tax of one dollar per year on each dentist represented. It is computed that there are twenty-two thousand dentists in the States to-day, and as, this year, twenty thousand dollars was paid into the treasury of the association, you see that the majority of the men are in societies. This alone is a great advance over your day, when the society men were a small minority."

"Undoubtedly. But, Doctor, is not twenty thousand dollars a small capital to buy patents with?"

"That is not an unnatural question; but the fact is, the association loses but very little on its patents; nothing, in fact. This brings us to your other question, the object of the joint convention of dentists and manufacturers. At this grand meeting, reports of all kinds are received. Working with a full knowledge thus obtained, the first thing done is to arrange a schedule of prices for the ensuing year."

"Do you mean to say that the dentist now has a say in the price of dental goods?"

"Assuredly. Is he not as much interested as the producer? The subject is thoroughly canvassed and the prices fixed."

"But suppose that the dental delegates should be in the majority and should fix the price so low that the manufacturers could not make a profit?"

"The dental delegates usually are in the majority, but they could not enact so arbitrarily, for the simple reason that as soon as too low a figure is placed on any special article, the manufacturers refuse to produce it. This is the salvation of the small dealers. There are many things placed so low that it would not pay for all the dealers to handle the article. Certain ones, therefore, declare that they will not enter into the competition on that line, when at once it becomes possible for a few dealers to make a profit by thus obtaining a sort of monopoly, even though the price is fixed by the convention.

"How do you compel the manufacturers to meet a required standard? Why could they not accept the price fixed by the convention and then supply an inferior article?"

"If such a thing could be proven the offending dealer could be expelled. But it is not probable. Wherever it is possible to have two or more grades in any product, prices are fixed for each grade. Thus there would be no temptation to make an inferior article, since it would at once come under a lower price. But now let me explain about the patents: All patents are presented to the convention and submitted to a committee of dealers. This committee reports on the probable demand for the new articles, the price at which they could be sold, and the royalty which the dealer would be willing to pay. On this basis a value is fixed for each patent, and that amount in cash is offered to the holder of the patent, who must in all cases be the inventor himself. If accepted, the patent passes to the Protective Association, who collects royalties from the dealers until the amount advanced has been recovered, with twenty per cent additional. As soon as this is accomplished the amount of the royalty is removed from the price of the article. Thus, you observe, that in the end the inventor is paid by the exact individuals who first use his invention. The dealer makes as much profit at the reduced price as before, the Protective Association merely advances the money.

"Suppose the inventor does not accept the offer, and sells his patent to one of the dealers?"

"In the first place, under the laws of the Trade Association, the buying of patents is prohibited. But, apart from that, no dealer will buy a patent when he cannot himself fix the price at which the article must be sold. No the only course open to the inventor would be to manufacture his article himself. Then, if he wished it sold by the Association dealers, he must submit to the rule of price as explained. Most men would prefer the certain cash."

"Since everything is so changed, tell me how are the journals conducted?"

"Properly speaking there is but one journal. There was much talk in your day against what was termed the

"trade journals;" but it was noticeable that the trade journals had the largest circulation. The reason was that the readers were as much interested in the developement of instruments as they were in the advancement of methods of practice. They as often read the advertisements as the other articles. To-day, therefore, the journalism of the profession is admirably systematized. Each State Society publishes the transactions of its meetings, and those of the local societies. These appear every quarter, the publication months being different in different States. There is but one general journal, which is managed jointly by the Protective Association and the Trade Association, profits or losses being equally divided. All dentists who are members of societies are compelled to subscribe, the price being two dollars per year. To men not members of societies the subscription price is double. All advertisments are paid for by the dealers. The journal publishes the best articles which appear in the society transactions. In this way the same thing is not told in a dozen ways, but the reader gets the kernel of wheat sifted from the mass of chaff. Besides, there are special articles written for the journal by the brilliant minds in the profession."

"Doctor, you have told me how the Protective Association buys patents. I suppose the twenty per cent profit on some is supposed to cover the loss on others. This being balanced, then what does it do with its money?"

"Besides meeting the current expenses of the Association, it offers awards to men who discover valuable facts in dental science, or who otherwise contribute to the general welfare of the profession in any shape where patent or copyrights do not offer a pecuniary return. It pays clinicians to make the circuit of the large cities and the colleges, and in various ways uses its capital for the advancement of dental science."

"I suppose there have been some changes in methods. I heard you say that Pyorrhœa is now very rare. In my time it was very common."

"Yes, but that is all changed. The advance made in Bacteriology early in this century made it evident that there was but one radical cure for so called constitutional diseases, and that was extermination."

"And have you accomplished that?"

"Not entirely, but we have made great progress. It was done in this way: When the National Marriage Law was passed, it provided that no one known to be affected with a constitutional disease should be granted a marriage license. All applicants, therefore, were compelled to pass a physical examination. That the law might not be too rigid at first, only a few of the worst diseases were placed on the list, such as scrofula, leprosy and syphilis. Later consumption and mental diseases were added, and from time to time the list was extended, until now, in the third generation since the passage of the law, we begin to recognize the wisdom of our forefathers. Naturally, pyorrhœa is not so prevalent."

"Do you still fill teeth with gold, or have you found something better?"

"That problem was solved by Erickson. About thirty years ago he offered, at one of the international dental conventions, a plastic material, which could be softened by heat and inserted as you did gutta-percha. It became about as hard as bone, and would receive a polish. It was adopted after thorough trial, and now, after thirty years, has proven to be the ideal filling material. It is manufactured now in a more excellent form than that made by Erickson, but is substantially the same. It is made in all shades, so that the teeth can be matched very accurately in color."

"Of what is it made?"

"The original formula of Erickson has been somewhat changed, but the principal ingredients remain the same. They are—"

At this moment there was a tremendous crash. People were thrown from their seats. I made a desperate effort to save myself, but—fell out of bed, wildly gripping the bed-clothing. The shock awoke me.

RODRIGUES OTTOLENGUI.

115 Madison Avenue, N. Y., January 1, 1890.

P. S.—Of course the above is suggested by Bellamy's book.—*Dental Review*. R. O.

